# A translational perspective on the anti-anhedonic effect of ketamine and its neural underpinnings

**DOI:** 10.1038/s41380-021-01183-1

**Published:** 2021-06-22

**Authors:** Erdem Pulcu, Calum Guinea, Philip J. Cowen, Susannah E. Murphy, Catherine J. Harmer

**Affiliations:** 1 Department of Psychiatry, University of Oxford, Oxford, UK; 2 Oxford Health NHS Foundation Trust, Warneford Hospital, Oxford, OX3 7JX UK

**Keywords:** Neuroscience, Psychology

## Abstract

Anhedonia, a pronounced reduction in interest or pleasure in any of life’s daily activities, is a cardinal symptom of major depression. In this *Perspective* article, we synthesise the recent evidence from rodent, monkey and human neuroimaging literature to highlight how the habenula, a small evolutionarily conserved subcortical structure located in the midbrain, may orchestrate the behavioural expression of anhedonia across fronto-mesolimbic networks. We then review how this circuitry can be modulated by ketamine, an NMDA receptor antagonist with rapid antidepressant properties. We propose that experimental paradigms founded in reinforcement learning and value-based decision-making can usefully probe this network and thereby help elucidate the mechanisms underlying ketamine’s rapid antidepressant action.

## Introduction

The discovery that a sub-anaesthetic dose of ketamine can have rapid antidepressant effects has been hailed as the most important discovery in 50 years of depression research [1, 2]. Ketamine is a high trapping, non-competitive NMDA receptor antagonist [3] and has been associated with rapid and reliable antidepressant effects in patients with treatment-resistant major depressive disorder (MDD), which emerge within hours and last for a number of days [4, 5]. Although these important clinical effects were discovered around two decades ago [6], an accepted mechanistic account of the antidepressant action of ketamine in depressed patients remains elusive. Recently, it was reported that a direct injection of ketamine into the lateral habenula (lHb) suppressed the burst activity of neurons in this region and produced a behavioural antidepressant effect in a rodent model of depression. This suggests that NMDA receptors play a role in the burst firing of the lHb neurons and raises the interesting possibility that ketamine might alleviate symptoms of depression via direct action on the lHb [7]. Ketamine is particularly shown to be effective in reducing anhedonia [5, 8].

Key work on understanding ketamine’s antidepressant drug actions have focused more on cellular mechanisms (for a review see [3]). In this *Perspective* article, we take a translational cognitive neuroscience approach and consider the potential mechanisms of ketamine across neural circuits important for reward and punishment. We present an overview of emerging evidence from rodent, monkey and human neuroimaging studies which cumulatively demonstrate how ketamine modulates the expression of anhedonic behaviours across habenula and fronto-mesolimbic networks.

## Fractionating anhedonia

Anhedonia is typically defined as diminished interest or pleasure in activities that people have previously enjoyed. Anhedonia can be operationalised into a set of subcomponents which recognise that anhedonic behaviour could be the result of dysfunction in several distinct processes, including the anticipation of, consumption of and learning about reward [9, 10]. These subcomponents serve as quantifiable outcome measures in preclinical and experimental medicine studies. Some of these outcome measures can be expressed by mathematical models of choice behaviour. Previous studies using computational modelling of human behaviour in signal detection tasks have dissociated differences between reward learning versus reward sensitivity (i.e., learning about rewards compared to the subjective value of the reward). Dysfunction in each one of these processes could contribute to anhedonia and related behaviours. In signal detection tasks, anhedonia in patients with depression has been shown to be selectively related to a reduction in sensitivity to reward (i.e. a reduction in the consummatory pleasure of reward) rather than differences in learning about reward from feedback [11, 12].

Distinct contributions of reward learning, anticipation, and consumption to the behavioural expression of anhedonia are also demonstrated in neuroimaging studies of reward processing. Auerbach et al. argued that different neuroimaging modalities may be optimised to study different subcomponents of anhedonia, recommending event-related functional magnetic resonance imaging (fMRI) of the basal ganglia for studying reward anticipation, and resting-state connectivity for reward consummation [9]. Although a previous large-scale study comparing 421 MDD patients with 488 healthy volunteers identified decreased resting-state functional connectivity with increasing depressive symptom severity in the medial orbitofrontal cortex [13], an area the authors highlighted as being commonly associated with reward processing, the degree to which this measure correlates with external measures of reward consummation remains an open question.

One important element of reward learning is the signalling of reward-prediction error (RPE), defined as the difference between an individual’s expectations about the outcome of an event and the actual outcome of said event (also see Box 1). Ground-breaking neurophysiology work in monkeys showed that activity in the ventral tegmental area (vTA) and the substantia nigra (i.e., structures within the basal ganglia) convey RPEs [14]. This role for basal ganglia structures has since been demonstrated in humans [15, 16] and has been identified as a neural signature of depression. For example, early fMRI studies in patients with MDD identified blunted temporal difference reward-learning signals in the ventral striatum (vSTR) [17], and diminished expected-reward value encoding in the amygdala-hippocampal complex [18]. A recent study [19] using the monetary incentive delay task further demonstrated the role of basal ganglia structures by showing that reward anticipation signals encoded in the nucleus accumbens were blunted over time. This signal was shown to be modulated by an interaction between anhedonia and response to positive mood induction, in a manner consistent with theoretical work suggesting that positive mood should reduce the magnitude of RPE by heightening subjective reward expectations [20]. Taken together, these studies demonstrate that basal ganglia structures encode RPEs during reinforcement learning (RL) and suggest a role for the basal ganglia in expression of anhedonia (for a more detailed consideration of clinical neuroimaging studies of reward processing see [21]. We think fractionating anhedonia into subcomponents will be important for understanding the specificity of treatment effects and ultimately improving the precision of treatment recommendations.

Box 1 Definitions of some key computational concepts mentioned in the manuscript • Reward prediction error is the difference between the agent’s expectations for an outcome of an event (i.e. what you predicted was going to happen) and the actual observed outcome of an event. The agent’s expectations are commonly estimated by reinforcement learning models such as the Rescorla-Wagner learning rule.

• Expected uncertainty relates to the known unreliability of predictive relationships in a familiar environment. For example, unsurprising fluctuations in day-to-day air temperature.

• Unexpected uncertainty relates to unsignaled shifts in context that result in highly unexpected outcomes, also known as environmental volatility. For example, a sudden peak in air temperature over and above seasonal average brought about by an hurricane.

• Information prediction error is the discrepancy between how informative a cue predicting an event is expected to be, and the degree to which it leads to a discernible relationship between [probabilistic] cues and outcomes in an environment. For example, the relationship between grey skies and subsequent rainfall. Cues become less informative when their predictive value falls from 100% to 50%, also known as maximum entropy.

## Interactions between habenula and dopamine neurons in reward and punishment processing

The habenula works in tandem with midbrain dopaminergic neurons during the processing of reward and punishment, particularly signalling prediction errors that arise when reward expectations are violated (i.e. the negative PEs) [22]. In monkeys, electrophysiological recordings have highlighted that the phasic activity of lHb neurons encodes *information* PEs (i.e., those arising from systematic manipulation of expected uncertainty (see Box 1), which determines the information content of outcomes during RL [23]) to a considerably greater extent than negative RPEs encoded in this region [24]. These studies suggest a role for the lHb as an “effective critic” of value-based decision-making through its reciprocal connections with the dopaminergic midbrain [24].

The habenula is an evolutionarily conserved brain structure which receives a broad range of inhibitory and excitatory inputs while providing a relatively small range of outputs [25, 26]. Areas projecting to the lateral aspect of the habenula (i.e., the lHb) contribute to a wide range of functions related to depression including circadian rhythm [27]; sleep; expression of negative emotions; stress and threat signalling; and motivated/appetitive behaviours [28–30]. Habenula and dopamine neurons undergo similar changes in firing rate pattern during the ramping up of tonic activity, and in their phasic responses at the initiation of reward-seeking behaviours [31]. While the tonic signal is thought to preferentially encode rewards, the phasic response occurs at the onset of both rewarding and punishing trials, reflecting the sensitivity of habenula and midbrain dopaminergic neurons to outcome salience irrespective of outcome valence [31]. This suggests that habenula and midbrain dopamine neurons and their connectivity are important for value and salience signalling, both of which may be relevant to anhedonia.

**Fig. 1:**
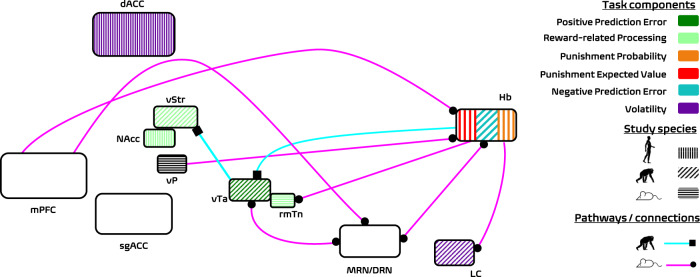
Schematic illustration of the regions implicated in anhedonia, the habenula’s functional connectivity and ketamine’s anti-anhedonic effect. In this schematic we collate evidence from key human [46, 50, 59, 83, 88, 89], monkey [14, 22, 24, 25, 31, 91] and rodent [7, 33, 34, 36, 37, 40] studies (depicted by angled lines on the surface of the coloured nodes with colours referring to different task components). The literature highlights the habenula as a key node in negative information processing, primarily through its direct and indirect (via MRN/DRN) connections to dopaminergic release sites as well as through input from mPFC. The majority of subcortical connections are based on findings from rodent studies (magenta coloured pathways) with some contributions from monkey neurophysiology (cyan coloured pathways). The direction of the connection between areas is represented by the location of the marker (e.g. on the line connecting the vP and the Hb, the circle is joined to the Hb to reflect that the vP provides input to the Hb). For clarity, a number of inputs to the habenula have been omitted (described in rodents and reviewed elsewhere [29]). Based on existing evidence in the literature, reversal learning tasks administered to human participants should probe the dACC and LC (key regions encoding environmental volatility); vTA, vSTR and Hb (key regions encoding positive and negative prediction errors) and regions of the mPFC encoding decision values and can be instrumental in understanding the effect of ketamine on subcomponents of anhedonia such as learning and reward processing.

An important excitatory input to the lHb is from the parvalbumin-positive neuronal populations [32] in the dopaminergic ventral pallidum (vP), which modulates analogues of depressive behaviours in rodent models [33]. Glutamatergic projections from the lHb to the rostromedial tegmental nucleus (rmTN) within the dopaminergic vTA have been shown to influence appetitive motivation and willingness to exert effort for reward in rodents [34]. Here, it is worthwhile to highlight that some of these studies [33, 34] rely on behavioural despair models (behavioural despair is defined as “a depression-like phenotype that reflects the feeling that nothing will improve” [34]) and assess anhedonic response in the forced swim test (FST), which is a commonly used outcome measure in preclinical research for antidepressant compounds [35]. Stimulation of the lHb to rmTN pathway is also shown to promote both active and passive types of behavioural avoidance in mice [36]. Neuroanatomic segmentation of the vTA identifies the rmTN at the tail end of this region, hosting populations of inhibitory GABAergic neurons which act as the “master brake” for the dopaminergic midbrain [37]. Optogenetic studies in rats have enabled the precise manipulation of the lHB to rmTN pathway that controls behaviours associated with anhedonia and a depressive phenotype. Stimulation of this pathway results in reduced mobility in the FST, and a reduction in the effort rats are willing to exert for reward [34]. The connections highlighted in this section are shown on a schematic diagram in Fig. 1.

## The habenula as a relay station regulating behavioural analogues of affective symptoms

Animal studies have highlighted a possible function for the habenula as a relay station orchestrating signals from both dopaminergic and serotonergic regions that regulate affective response during reward-guided interactions with the physical environment. Two connections that are important for this function are the lHb inputs to the dorsal raphe nucleus (dRN) and the medial raphe nucleus (mRN) [25, 29]. The dRN receives both direct (glutamatergic) and indirect (via rmTN) projections from the lHb, this indirect pathway provides feedforward inhibition to serotonergic (5-HT) neurons. A previous rodent study showed that glutamate signalling contributes to the learning of reward associations [38], this is compatible with recent learning theories which posit that receipt of unexpected rewards during learning should uplift mood and attenuate the inhibitory influence of the lHb on the dRN via the direct pathway [29]. The dRN pathway would therefore be a candidate route via which the lHb neurons can integrate affective information (e.g., emotional response to reward outcomes) into reward processing [39], with causal implications for depressed and elated mood through aberrant learning from negative RPEs [20]. It is possible that impairments in learning about the reward contingencies in the environment which would lead to heightened negative RPE signalling in the habenula may be depressogenic. Intriguingly, both the lHb and dRN receive projections from the mPFC and optogenetic manipulation of these projections reduced mobility in the FST, highlighting a potential mechanism for depressive behaviours embedded in this circuitry [40]. These studies also suggest a translational hypothesis for future neuroimaging studies in which the activity of the human habenula might be parametrically modulated by momentary mood responses to reward outcomes received from the environment [41]. The connections highlighted in this section are also shown in Fig. 1.

## The role of the habenula in anhedonia and depression

Thus far, we have reviewed evidence that describes the habenula’s role in the processing of reward and punishment as well as its contribution to analogues of depressive behaviours, mostly based on findings from rodent studies [7, 34, 42–44]. One intriguing finding from the rodent literature demonstrated that acute stress can reverse the activity of reward selective neurons in the lHb [42], such that they respond to rewards as though they were punishing. Considering the evolutionarily preserved role of the habenula across species, this reversal in activity which is accompanied by an increase in the magnitude of responses to reward omission, provides a candidate mechanism through which exposure to stress can trigger the onset of anhedonic behaviours. Whilst the human habenula’s functional connectivity with midbrain dopaminergic areas such as the vTA, as well as serotonergic areas such as the dRN [45] and general anatomical parcellation are well-characterised [42–44], relatively few studies have assessed whether the habenula makes a similar contribution to depressive symptoms in humans.

A few studies investigating the habenula’s function in humans have provided complementary evidence to findings shown in rodent models. One of these human studies used a Pavlovian conditioning paradigm in which abstract fractals were probabilistically associated with different types of outcomes: electroshocks or monetary [win/loss] outcomes [46]. In healthy controls, the habenula responded positively to the expected values of cues predicting a shock but did not encode the expected value of monetary outcomes. These findings are in line with an earlier monkey neurophysiology study which suggested that during Pavlovian conditioning the habenula is most responsive to the expected value of the worst outcome in the environment (e.g. least rewarding or frequently punishing outcomes) [47].

In a follow-up study, the same group of authors reported that this habenula response is sensitive to severity of self-reported depressive symptoms in nonclinical volunteers, such that a subset of participants who reported higher depressive symptoms did not show a positive habenula response to shocks. Furthermore, a group of patients with MDD showed a deactivation in the same region, leading to significant differences between patients and non-depressed healthy volunteers [48]. Interestingly, this deactivation to shock-predicting cues in people with depression is similar to response of the habenula to rewards in healthy volunteers who participated in the same group of author’s previous work [46], as well as in monkeys [14]. Notably, the habenula response seen in healthy controls was positively correlated with a measure of conditioned suppression (in this case, slower response times in a flicker detection task completed on shock trials, which were also correlated with pupil dilation) [46]. The authors suggest the positive habenula response to shocks seen in control participants may potentiate avoidance behaviour; the negative response observed in MDD patients therefore may result in a loss of the capacity for active avoidance [48]. Overall, these studies outline a dual function for the habenula in the anticipation and experience of rewards and punishments relevant for understanding cognitive processes that are impaired by symptoms of MDD.

Consistent with the findings from monkey neurophysiology studies [24, 49], functional neuroimaging suggests that the human habenula also encodes the expected values of negative outcomes during value-based decision-making. For example, the habenula has been shown to respond to monetary loss outcomes to a larger extent than win outcomes, and this activity is parametrically modulated by the probability of losses [50]. Interestingly this pattern of activity was not seen in patients with MDD, suggesting that depression may impair a core function of the habenula that is, encoding the expected value of negative outcomes. Taken together, these studies demonstrate that the habenula functions similarly in humans and laboratory animals during reward and punishment processing and how this function is influenced by symptoms of depression.

## Loci of ketamine’s antidepressant action

In this section, we will highlight the role of brain regions that play a key role in ketamine’s anti-anhedonic effects in preclinical models. As we highlighted in the introduction, the lHb has recently been identified as an important site for ketamine’s rapid antidepressant action [5]. Optogenetic stimulation of lHb neurons results in a burst firing pattern that underlies an expression of anhedonia and behavioural despair in rodents [7]. This study also demonstrated that ketamine attenuates the burst firing of habenula neurons, in turn relieving rodent analogues of depressive symptoms.

The other key region involved in ketamine’s antidepressant action in rodent models of depression is the medial prefrontal cortex (mPFC), which notably projects to the habenula [40], and involves an increase in the extracellular excitatory glutamate levels in this area [51]. Chronic unpredictable stress, which is an experimental model of depression in rodents, is linked with reduction in the number of synapses in mPFC [52], and subsequent studies have shown that ketamine infusion can reverse synaptic deficits and an anhedonic behavioural response caused by chronic exposure to stress [53]. Recent advances in positron emission tomography may be useful in translating these studies, to aid the identification of similar changes in mPFC synaptic architecture in MDD patients who are treated with ketamine infusion relative to their baseline (i.e. before treatment onset in a within-subject design) [54].

Another rodent study demonstrating a role for the mPFC used an affective bias model of depression and compared ketamine with a traditional antidepressant (venlafaxine) [55]. Negative bias was induced by administering a benzodiazepine receptor inverse agonist (acting as an acute stressor in this rodent model) prior to one-shot learning via which rodents encoded the subjective value of primary rewards (i.e., a food substrate). Although one might argue that negative bias models of depression relate to depressive symptoms which are distinct from anhedonic behaviours, the experimental approach used by this study relied on probing components of cognition such as reward processing and learning which also relate to anhedonic behaviours. Stuart et al. showed that rapid and delayed action antidepressants act via distinct neuropsychological mechanisms [55], and demonstrated that ketamine engages the mPFC in rapidly mitigating the effect of negative bias associated with a reward substrate conditioned under an acute stressor.

The evidence reviewed in this section highlights that ketamine acts on both cortical and subcortical structures, specifically the mPFC and lHb. Action at both of these sites rapidly mitigates analogues of depressive symptoms in rodent models.

## NMDA modulation of habenula neurons might be orchestrating the expression of anhedonia across fronto-mesolimbic and fronto-striatal networks

So far, we have synthesised findings from key animal experimental and neuroimaging studies of the habenula which have focused on its functional relationship with striatal dopaminergic regions during RL, and with other key neurotransmitter hubs within the mesolimbic network [56] involved in the expression of anhedonic and other depressive behaviours [40]. We have highlighted that optogenetic manipulation of axons projecting from the mPFC to the lHb regulate analogues of depressive behaviours in rodents [40]. Furthermore, we have explained how direct infusion of ketamine to the lHb [7] or the mPFC [52] can attenuate analogues of depressive symptoms. These findings suggest a potential mechanism through which mPFC may regulate subcortical contributors to the depression phenotype. In this section, we will expand on the role of the mPFC and highlight findings from monkey neurophysiology and human neuroimaging studies. Here, it is worthwhile to highlight the importance of drawing inferences from monkey neurophysiology as a bridge between the preclinical studies that we reviewed in the preceding sections and human neuroimaging [57]. Although the functional neuroanatomy of habenula is assumed to be preserved across species, more translational work is needed to understand the degree to which prefrontal cortex maintains a similar functional homology across rodents, monkeys and humans.

The use of multivariate approaches in human fMRI has enabled measurement of the heterogeneity in responses within the mPFC, the ventral sections of the mPFC in particular are consistently implicated in the relative coding of value in humans [58, 59] and monkeys [49]. Several human neuroimaging studies suggest that using prosocial incentives can effectively probe regions of the vmPFC during social learning [60] and decision-making [61]. For example, a recent study suggested that depressive symptoms correlate with a heightened neural response to negative delayed social incentives in the subgenual [cingulate] cortex [62] (sgACC, Brodmann area (BA) 25 [63], as well as the posterior-most section of the mPFC overlapping with BA32 and parts of BA24 and BA33 residing below the genu i.e. “the knee” of the corpus callosum). It is noted that these cortical areas may not be structurally [64] or functionally homologous in the context of reward processing [65].

The sgACC, broadly defined based on the Brodmann areas highlighted above, has been consistently implicated in the aetiology of depression [66–69], prediction of its recurrence [70] and treatment [71]. Ketamine’s antidepressant effect has been shown to depend on the expression of the neuropeptide precursor VGF in the sgACC, which is significantly depleted in patients with MDD, as demonstrated by post-mortem examinations, while its overexpression is shown to promote resilience to chronic stressors in a mouse model [72]. In lower primates such as marmoset monkeys, experimentally induced overactivity of the sgACC is associated with an anhedonic anticipatory response to primary rewards and reduced willingness to exert effort to obtain reward, both of which can be ameliorated by acute peripheral administration of ketamine but not by traditional antidepressants such as citalopram [73]. Remarkably, across species, the role of sgACC appears to be selectively related to anticipatory responses, rather than consummatory responses. Using decision-making probes of the sgACC in human neuroimaging studies of participants who have received a ketamine infusion may be a useful avenue in testing a translational prediction that ketamine should modulate reward processing impaired by anhedonia, such as value encoding.

Human neuroimaging studies also demonstrated ketamine related changes in functional connectivity across the fronto-striatal circuitry. For example, a recent study showed increased functional connectivity between the caudate and PFC regions (dlPFC and vlPFC), and between the putamen and PFC regions (more specifically perigenual ACC and the orbitofrontal cortex) in treatment-resistant depression (TRD) patients [74]. However, in healthy volunteers, ketamine reversed these functional connectivity effects. In addition, there is preliminary evidence to suggest that improvements in depressive symptoms following ketamine infusion in patients with MDD correlates with an increased functional connectivity between the right habenula and right frontal pole [75].

Taken together, the studies that we reviewed in this section suggest that reward circuitry distributed across fronto-mesolimbic and fronto-striatal networks are important in understanding ketamine’s antidepressant effect. A comprehensive understanding of ketamine’s action would require studying its direct effects on reward processing (e.g., in healthy volunteers) as well as its indirect effects on reward processing through an action on anhedonia (e.g., in patients with depression). However, there are not any studies to our knowledge, which have investigated the direct effects of ketamine on reward processing in humans or monkeys. On the other hand, only a limited number of rodent studies have investigated how ketamine modulates reward processing within a temporal discounting framework. This is a relevant domain as previous literature suggests that temporal discounting decisions are also impaired in patients with depression [76, 77] who display a higher discounting tendency associated with a preference for immediately available rewards. However in rodents, a clinically relevant sub-anaesthetic dose of ketamine (5 mg/kg) did not reveal consistent results, one study suggesting an increase in discounting [78], whereas a more recent study did not show any significant changes in discounting behaviour at this dosage [79]. This limited literature suggests that more studies are needed across all species to be able to decompose ketamine’s direct and indirect effects on reward processing.

## Experimental probes and future directions

In this *Perspective* article, we have reviewed evidence showing that habenula and midbrain dopaminergic neurons work closely in signalling reward and punishment PEs during tasks performed over a single testing session (i.e., technically described as “online learning” [80]), and that these neural processes are likely to be involved in the neural basis of anhedonia. We have also highlighted evidence that ketamine produces its antidepressant effects through action on the habenular complex and fronto-mesolimbic networks. As we are entering the third decade of human research into the antidepressant effects of NMDA receptor antagonists, future studies could harness existing computational modelling, functional connectivity, novel neuroimaging analysis methods and imaging modalities with higher spatial and temporal resolution (e.g., scan human participants at higher magnetic field strengths [81, 82] or with MEG and pupillometry). So far only a small number of studies have focused on the human habenula in healthy and clinical populations. The current state of the human neuroimaging literature does not reflect the richness of animal models describing the mechanisms of antidepressant action across the habenular complex and fronto-mesolimbic circuitry (Fig. 1). In this section we will elaborate on experimental methods that can probe these systems in human participants and help bridge this translational gap.

Although both Pavlovian and instrumental conditioning frameworks have been adopted by previous studies, RL tasks involving reversals in which the better option in the environment changes at time points unknown to the participant, might be the best experimental approach to probe these systems. Continuous reversals in the task environment mean that the participant will continue experiencing PEs over the course of the experiment, resulting in continuous engagement of the subcortical structures implicated in RL. In RL terminology, unexpected uncertainty arising from dynamically changing outcome contingencies is known as volatility [83] (Box 1). It is important to highlight that awareness of environmental volatility relates to a second-order statistic which builds up as participants experience more trials during the task (i.e., learning about the structure of the environment). Seminal work in this area conducted by Behrens and colleagues demonstrated that dorsal anterior cingulate cortex (dACC) encodes volatility signals (i.e., the rate with which the environment changes) during reversal learning of non-social and social reward contingencies [83, 84]. In similar reward-learning tasks, regions of the dACC are also shown to encode trialwise PE estimates in both humans [85] and monkeys [49]. Recent electrophysiology work conducted in patients with pharmacologically intractable epilepsy has extended these findings, suggesting that neuronal populations within the dACC respond more selectively to punishments than to rewards [86]. This is in line with previous findings from monkey electrophysiology revealing similar results that, demonstrate the within-trial temporal frame of the neural activity across dACC and habenula neurons, where the latter are shown to be quicker to respond to omission of reward [87]. Across trials, the dACC is shown to be engaged with the running history of outcomes, consistent with its role in encoding volatility signals, whereas habenula neurons were involved more strongly in guiding behavioural adjustments in response to the outcome of the current trial [87], potentially indicating a role in the implementation of learning rates. Therefore, it is highly likely that adjustment of learning rates to environmental volatility/uncertainty, a key cognitive process with survival value across the animal kingdom, might be implemented across dACC and habenula neurons. Considering that the habenula has influence over the neurotransmitter hubs of the mesolimbic pathway and these projections extend to prefrontal cortex and the dACC, it is possible that a complete feedback loop exists between the fronto-mesolimbic pathway and the habenula, though this proposed link remains to be explored by future human and monkey neurophysiology studies. This feedback loop may rely on key cognitive processes implemented across dACC and habenula neurons and is likely to be critical for understanding the neurobiology of depression (Box 2).

In human physiology studies, the activity of the pupil-linked arousal systems is shown to encode the volatility of punishment contingencies [88] more strongly than the volatility of reward contingencies [89]. A number of previous studies have demonstrated that changes in pupil dilation relate to the activity of central norepinephrine neurons [90, 91]. Both the dACC and the central norepinephrine system are also important target sites for understanding ketamine’s antidepressant action. For example, previous work suggests that the degree of ketamine’s anti-anhedonic effect correlates with increased glucose metabolism in the dACC [8] and the habenula, a key subcortical target of injection for ketamine’s antidepressant effects in rodents, is also known to exert control over the locus coeruleus and the central norepinephrine system [26, 92, 93]. These areas may also be linked in terms of the cognitive processes they encode during reward learning. For example, the central norepinephrine system (as indexed by pupil dilation) responds to informative/volatile outcomes in the environment and the habenula encodes information PEs in monkeys. An additional strength of probing these systems with reversal learning tasks is that the computational neuroscience literature holds a number of well-established [83] and emerging [23, 85, 94] mathematical models (i.e., Bayesian and RL) which can be instrumental in revealing precisely how ketamine modulates computational processes such as learning rates and reward/punishment sensitivity across the habenular complex and the fronto-mesolimbic networks (Fig. 1). Indications from the rodent pharmacology studies are that ketamine may affect sensitivity to reward magnitude, plausibly reflecting one of the aforementioned components of anhedonia.

We believe that converging evidence presented in this manuscript from existing rodent and monkey models warrants an interdisciplinary approach for the next decade of human research, designed to translate these insights into tractable models of rapid antidepressant action in depressed patients and thereby produce a radical improvement in the management of clinical mood disorders. Moreover, given anhedonia is a transdiagnostic feature of many psychiatric and neurological conditions, the translational approach we outlined here may have utility for clinical improvement in all neuropsychiatric conditions in which anhedonia manifests.

Box 2 Summary and future directions • The habenula, a subcortical brain structure that is evolutionarily preserved across species, orchestrates behavioural expression of anhedonia across fronto-mesolimbic and fronto-striatal networks.

• Translational evidence suggests that ketamine, a noncompetitive NMDA receptor antagonist with a rapid antidepressant/anti-anhedonic profile that is consistently observed in patients with depression, modulate neural underpinnings of anhedonic behaviours in these circuits, acting on the habenula, dACC, mPFC and sgACC.

• Reversal learning paradigms which capture learning and reward/punishment processing domains of anhedonia can effectively probe the dACC, vmPFC, vSTR and habenula simultaneously, and elucidate mechanisms of ketamine action.

• Overall human neuroimaging studies contributing to our understanding of these circuits are lagging behind animal studies in terms of quantity. Future studies employing neuroimaging methods with higher temporal and spatial resolution are needed to develop a better understanding of ketamine’s anti-anhedonic action.
